# Genetic and non-genetic factors in prediction of early pubertal development in Chinese girls

**DOI:** 10.3389/fendo.2024.1413528

**Published:** 2024-07-01

**Authors:** Weiqin Li, Yuexin Du, Lingyan Feng, Panpan Song, Leishen Wang, Shuang Zhang, Wei Li, Dandan Zhu, Huikun Liu

**Affiliations:** ^1^ Institute of Maternal and Child Health, Tianjin Women and Children′s Health Center, Tianjin, China; ^2^ Child Health Care, Tianjin Women and Children′s Health Center, Tianjin, China; ^3^ School of Public Health and health sciences, Tianjin University of Traditional Chinese Medicine, Tianjin, China; ^4^ Disease Screening Center, Tianjin Women and Children′s Health Center, Tianjin, China

**Keywords:** early pubertal development, girls, environmental factors, genetic factors, prediction model

## Abstract

**Objective:**

The objective of this study is to develop a combined predictive model for early pubertal development (EPD) in girls based on both non-genetic and genetic factors.

**Methods:**

The case-control study encompassed 147 girls diagnosed with EPD and 256 girls who exhibited normal pubertal development. The non-genetic risk score (NGRS) was calculated based on 6 independent biochemical predictors screened by multivariate logistic regressions, and the genetic risk score (GRS) was constructed using 28 EPD related single-nucleotide polymorphisms (SNPs). Area under receiver operator characteristic curve (AROC), net reclassification optimization index (NRI) and integration differentiation index (IDI) were used to evaluate the improvement of adding genetic variants to the non-genetic risk model.

**Results:**

Overweight (OR=2.74), longer electronic screen time (OR=1.79) and higher ratio of plastic bottled water (OR=1.01) were potential risk factors, and longer exercise time (OR=0.51) and longer day sleeping time (OR=0.97) were protective factors for EPD, and the AROC of NGRS model was 83.6% (79.3-87.9%). The GRS showed a significant association with EPD (OR=1.90), and the AROC of GRS model was 65.3% (59.7-70.8%). After adding GRS to the NGRS model, the AROC significantly increased to 85.7% (81.7-89.6%) (*P*=0.020), and the reclassification significantly improved, with NRI of 8.19% (*P*= 0.023) and IDI of 4.22% (*P <*0.001).

**Conclusions:**

We established a combined prediction model of EPD in girls. Adding genetic variants to the non-genetic risk model brought modest improvement. However, the non-genetic factors such as overweight and living habits have higher predictive utility.

## 1 Introduction

Early pubertal development (EPD) is commonly defined as the development of secondary sexual characteristics of girls before 8 years old and boys before 9 years old (1). In recent years, the prevalence of EPD has shown an increasing trend. An observational study from the United States showed that 10% of white girls and 23% of black girls at the age of 7 years old have started puberty (2). According to a school-based survey in China, 11.47% of girls before age 8 and 3.26% of boys before age 9 had signs of EPD (3), with a higher incidence in girls than in boys. EPD will lead to accelerated skeletal maturation, advanced bone age, and early epiphysis closure, all of which will affect the final adult height. In addition, it may result in psychological issues or abnormal social behavior (4). Early menarche in girls is also related to long-term health consequences including obesity, type 2 diabetes, breast cancer, and cardiovascular events (5). Therefore, EPD has attracted the attention of global public health concerns, and it is very important to evaluate and diagnose girls suspected of EPD in time.

The timing of normal pubertal onset varies greatly and is influenced by environmental and genetic factors. It is considered that overnutrition, insufficient exercise, insufficient sleep (6), and expose to some endocrine-disrupting chemicals (EDCs) (7) are linked to early puberty. Currently, there are no risk prediction models for EPD developed worldwide, and the prediction of EPD risk has not been part of routine practice for families.

In addition to environmental factors, many studies have examined the relationship between candidate genes and pubertal timing. Mutations in Delta-like homolog 1 (DLK1), the kisspeptin system (KISS1 and KISS1R), and the Makorin RING-finger protein 3 (MKRN3) gene have been identified in sporadic and familial cases of central precocious puberty (8, 9). Besides, some large-scale genome-wide association studies (GWAS) have begun to search for single-nucleotide polymorphisms (SNPs) that are associated with the onset time of puberty (10). One single SNP has a low explanation ability in disease risk, but these susceptibility loci in combination could explain more disease risk variation. Until now, there is no research on constructing a genetic risk score based on the known EPD risk alleles.

The aim of this study was firstly to establish a non-genetic risk model to predict EPD in girls, and then assess the predictive ability improvement as well as the reclassification of adding genetic variants to the non-genetic risk model.

## 2 Methods

### 2.1 Study design and participants

This was a case-control study conducted between October 2019 and August 2022 in Tianjin Women and Children’s Health Center, China. It encompassed 147 girls who were newly diagnosed with EPD and 256 girls who exhibited normal pubertal development. The inclusion criteria of case group were: 1) Girls. 2) Confirm the Chinese diagnostic criteria for EPD in girls (11), which involves the development of secondary sexual characteristics before the age of 8 or menstruation before the age of 10. All girls were physically examined by qualified female pediatricians in a private room, and then were given a B-ultrasound test of breast, uterus and ovary using a Philips EPIQ7 ultrasonic instrument. The Tanner Staging was used to evaluate children’s secondary sexual characteristics (12, 13). 3) Children and their guardians agreed to participate in the study and signed the informed consent form. Exclusion criteria include: 1) Secondary central precocity, such as central nervous system occupying, postoperative, infection, trauma, chemotherapy or radiotherapy, and congenital dysplasia. 2) Other primary diseases that may lead to EPD, such as McCune-Albright syndrome, congenital adrenal hyperplasia, granulomatous disease, congenital hypothyroidism, cerebral palsy, and malignant tumor. 3) A history of using hormone drugs.

Based on the routine health examination of kindergartens/primary school students in Tianjin, 256 girls of the same age (7.72 ± 1.0 vs. 7.60 ± 1.0 years) who exhibited normal pubertal development were recruited as the control group. The inclusion criteria for the control group were: 1) With normal pubertal development, and without secondary sexual development before the age of 8. All girls were physically examined by qualified female pediatricians in a private room, and Tanner Staging method was used to evaluate children’s secondary sexual characteristics. 2) Children and their guardians agreed to participate in the study and signed the informed consent form. Exclusion criteria include: 1) Primary diseases that may lead to EPD, such as McCune-Albright syndrome, congenital adrenal hyperplasia, granulomatous disease, congenital hypothyroidism, cerebral palsy and malignant tumor. 2) A history of using hormone drugs.

This study was approved by the Ethics Committee of Tianjin Women and Children Health Center (approval number: ky-20190119), and all guardians of the research subjects have signed the informed consent form.

### 2.2 Data collection methods

The weight, height, and waist circumference of children were measured according to standardized procedures. Using a digital scale (TCS-60, Tianjin Weighing Apparatus Co., China), body weight was measured to the nearest 0.01 kg. Using a station meter (SZG-180, Shanghai Zhengdahengqi, China), standing height was measured to the nearest 0.1 cm. Children’s waist circumference was measured midway between the lower rib margin and the iliac crest, and the measurement was accurate to the nearest 0.1 cm. A body composition analyzer (Inbody J-20, Korea) was used to measure the body fat percentage of children. Body mass index (BMI) was calculated by dividing weight (kg) by the square of height (m). According to the WHO age- and gender-specific growth reference of 0~60 months (14) and 5~19 years old (15), children’s Z scores of BMI for age were calculated. Overweight/obesity in children was defined as BMI ≥ 85^th^ percentiles (Z score of BMI for age ≥ 1.035) which included both overweight and obesity to improve the statistical power of test.

We obtained the information of the participants through a questionnaire survey. The researcher filled out the questionnaires during a face-to-face interview with children and their guardians. The questionnaire consisted of five sections: 1) General information (date of birth, age, and ethnicity); 2) Parents’ information (parents’ weight and height, mother’s first menarche age, and father’s first spermatogenesis age); 3) Diet information for the previous month and the average per day was calculated, by asking “How often did you eat snacks, including chips, cookies, candy, cake, chocolate, ice cream and other desserts?”, “How many eggs did you eat, including all kinds of cooking like poaching, frying, and being included in other foods?”; 4) Behavior information in 1 month prior to the survey, and the average time per week was calculated, including: electronic screen watching time, moderate-to-vigorous physical activity time (MVPA) time, and also time spent on the roadside where children might be exposed to automobile exhaust (including walking, biking or parking on the road or roadside); the proportion of plastic bottled water in total drinking water was also investigated. Moderate physical activity is defined as 12-14 of 20 grades in the RPE scale (16), and the intensity is 3.0 to 5.9 metabolic equivalent (MET), such as skating, jogging, cycling at normal speed, etc. Vigorous physical activity is defined as 15 or above of 20 grades in the RPE scale, and the intensity is ≥ 6 MET, such as running fast, carrying heavy objects, cycling fast, etc; 5) Sleep habits in 1 month prior to the survey, and the average time per day was calculated, including: bedtime at night, wakeup time in the morning, average time of day sleeping and average time of night sleeping. The total sleeping time was obtained by night sleeping time plus day sleeping time.

#### 2.2.1 SNPs selection and genotyping

The buccal mucosa cells of children were collected using two buccal swabs by trained investigators according to the manufacturers’ instructions. According to the manufacturer’s instructions (Epicentre Biotechnologies, Madison, WI), DNA was extracted from two buccal swabs (placed in the same test tube) by the heat lysis method. SNP typing was performed by MassARRAY flight mass spectrometry. The success rate of genotyping was >98%. In order to control the quality, 10% of the samples were re-genotyped, and the coincidence rate was >99%.

Mutations in the MKRN3 gene, the kisspeptin system (KISS1 and KISS1R), and DLK1 have been identified in sporadic and familial cases of central precocious puberty (9), and 13 SNPs were selected from these genes. Another 15 highly correlated SNPs were selected from the GWAS conducted in East Asia (10). The SNP, reported gene, functional class, and alleles are presented in **Table 1**.

**Table 1:** List of the 28 single nucleotide polymorphism and their association with early puberty development.

No.	Reported gene	SNP	Functional class	Alleles	MAF (%) CHB	OR 95% CI
SNPs from candidate genes associated with central precocious puberty						
1	KISS1	rs4889[33]	Exon-missense	G/C	35.9	**1.54 (1.15-2.06)**
2	KISS1	rs71745629	frame shift	T/DEL	34.5	**1.50 (1.12-2.01)**
3	KISS1	rs3924586	promoter	C/T	33.5	1.30 (0.94-1.78)
4	KISS1R (GPR54)	rs350132[34]	Exon-missense	A/T	22.8	1.17 (0.86-1.58)
5	KISS1R (GPR54)	rs10407968	Exon-synonymous	A/G	14.6	1.07 (0.68-1.67)
6	DLK1	rs28362569	promoter	CCCC/CCC	43.2	1.02 (0.76-1.37)
7	DLK1	rs17099637	promoter	C/T	27.7	1.01 (0.73-1.40)
8	DLK1	rs876374	3’UTR	C/A	21.8	1.14 (0.81-1.62)
9	DLK1	rs1555406	3’UTR	C/T	14.1	1.19 (0.80-1.76)
10	MKRN3	rs12441827[35]	promoter	T/C	36.9	1.13 (0.85-1.50)
11	MKRN3	rs34389827	promoter	AAAAAAAA/AAAAAAA	36.4	1.11 (0.84-1.48)
12	MKRN3	rs12148769[36]	intergenic	G/A	31.1	1.06 (0.78-1.43)
13	MKRN3	rs12439354	5 near	A/G	36.9	1.12 (0.84-1.49)
The top 15 SNPs from the GWAS conducted in East Asia						
14	intergenic	rs79195475	intergenic	T/C	20.9	1.00 (0.72-1.39)
15	intergenic	rs1023935	intergenic	T/C	9.2	1.17 (0.76-1.82)
16	SATB2	rs1400974	promoter	G/A	40.8	1.20 (0.89-1.61)
17	IGSFI1	rs11715566	intergenic	T/C	48.1	1.24 (0.94-1.65)
18	LIM28B	rs2153127	intergenic	C/T	38.8	1.23 (0.92-1.65)
19	LIN28B	rs7759938	intergenic	T/C	28.2	1.05 (0.75-1.48)
20	TMEM38E	rs10453225	intron	T/G	48.1	1.21 (0.91-1.61)
21	TRPC6	rs10895140	intron	A/G	45.6	1.11 (0.85-1.45)
22	MKL2	rs246185	intron	C/T	49.5	1.03 (0.78-1.36)
23	KCTD13	rs1129700	intron	T/C	41.7	1.29 (0.96-1.72)
24	DLGAPI	rs12607903	intron	C/T	49.5	1.13 (0.89-1.43)
25	KDM4A	rs2274465	intron	C/G	24.8	1.18 (0.87-1.61)
26	RXRG	rs466639	intron	C/T	11.7	1.01 (0.68-1.50)
27	ZNF483	rs10980921	intergenic	T/C	24.8	1.25 (0.88-1.78)
28	CADMI	rs11215400	intron	A/C	12.1	**1.68 (1.06-2.68)**

Bold value means P<0.05.

#### 2.2.2 Score construction

We calculated a genetic risk score (GRS) with the selected 28 SNPs. Logistic regression was conducted to determine the association between the number of risk alleles and EPD. The weighted GRS was computed by multiplying the number of risk alleles (0, 1, or 2) of each SNP by the natural logarithm of OR in the Logistic regression for that allele and summing across all SNPs. Similarly, the calculation principle of the weighted non-genetic risk score (NGRS) was the same as that of GRS. For each individual, the NGRS was calculated by the sum of risk factors weighted by OR (*β*) values of different non-genetic risk factors in Logistic stepwise regression. Assuming that genetic and non-genetic factors were independent, we added the weighted GRS to each risk algorithm to obtain a combined genetic and non-genetic score (CRS).

#### 2.2.3 Reclassification of EPD risk

We used the net reclassification improvement (NRI) and integrated discrimination improvement (IDI) to quantify the extent to which CRS moved people to risk categories that better reflected their event status (17, 18). Similar to NRI, IDI large than zero reflected a positive improvement, meaning that the prediction ability of the new model was improved compared with the old model. We used both categories (EPD risk: < 10%, 10% - <90%, and ≥ 90%) and continuous EPD risk to calculate NRI. Girls with EPD were considered to be correctly reclassified if they moved to a higher risk category, while those who moved to a lower risk category were considered to be incorrectly reclassified. Girls without EPD were the opposite. We also used continuous EPD risk to calculate NRI, which reflected the change defined by any upward or downward change of the specified risk.

### 2.3 Statistical analyses

The general characteristics between groups were compared by using *Chi-square* test for categorical variables, and student’s *t*-test for continuous variables if their normal distribution was not rejected. The prediction models were constructed by logistic regression analyses. The area under receiver operating characteristics curve (AROC) was utilized to evaluate the predictive abilities of the GRS, NGRS and CRS models, and the DeLong test was used to compare the AROC values of different models (19).

A family history of diseases, in specific cases, reflects genetic predisposition, so there is a strong association between the family history of EPD and EPD related GRS. In addition, family disease history can share environmental and lifestyle factors, and even inclusive fetal programming. Therefore, we included two models when constructing NGRS and CRS: Model 1, without mother’s first menarche age or father’s first spermatogenesis age; Model 2, with mother’s first menarche age and father’s first spermatogenesis age.

The criterion of statistical significance was < 0.05 (for two-sided tests). All statistical analyses used IBM SPSS Statistics 24.0 (IBM SPSS, Chicago, IL) and R 4.0 (R Foundation for Statistical Computing, Vienna, Austria) software programs.

## 3 Results

### 3.1 General characteristics

The baseline characteristics of the participants were showed in **Table 2**. The age of girls in the case group and the control group were 7.72 ± 1.0 years and 7.60 ± 1.0 years, respectively, and there was no statistical difference between the two groups (*t*=-1.300, *P*=0.194). There were significant differences in children’s weight, height, waist circumference, BMI, Z scores for BMI, MVPA, electronic screen time, sedentary time, the proportion of plastic bottled water, time on the roadside, bedtime at night, day sleeping time, night sleeping time, and total sleeping time (all *P* values <0.05).

**Table 2:** General information for the case and control groups.

	Case group (n=147)	Control group (n=256)	*t*/χ^2^ value	*P* value
Age (years)	7.72 ± 1.0	7.60 ± 1.0	-1.300	0.194
Information of parents				
Mother’s age (years)	36.39 ± 3.8	36.72 ± 4.7	0.778	0.437
Mother’s BMI (kg/m^2^)	23.80 ± 3.9	23.32 ± 3.9	-1.194	0.233
Mother’s age of menarche (years)	12.24 ± 1.4	12.82 ± 0.8	4.608	<0.001
Father’s age (years)	37.47 ± 4.5	37.98 ± 4.9	1.059	0.290
Father’s BMI (kg/m^2^)	25.86 ± 3.4	26.3 ± 4.5	1.126	0.261
Father’s age of first spermatogenesis (years)	13.69 ± 1.1	14.20 ± 1.2	4.254	<0.001
Physical examination				
Height (cm)	132.06 ± 10.2	126.35 ± 7.8	-5.854	<0.001
Weight (kg)	31.21 ± 9.4	26.60 ± 6.7	-5.213	<0.001
BMI (kg/m^2^)	17.57 ± 3.3	16.47 ± 2.7	-3.424	0.001
Z scores for BMI	0.69 ± 1.4	0.26 ± 1.2	-3.264	0.001
Waist circumference (cm)	62.47 ± 7.7	56.66 ± 6.8	-7.874	<0.001
Body fat percent (%)	22.72 ± 6.6	22.33 ± 4.1	-0.660	0.510
Overweight^*^	60 (40.8)	62 (24.2)	12.187	<0.001
Diet information				
Eggs ≥ 250 g/week, n (%)	89 (70.1)	163 (65.7)	0.722	0.395
Sweet snacks ≥ 3 times/week, n (%)	94 (74.0)	197 (79.4)	1.419	0.234
Behavior information				
Electronic screen time (hours/day)	1.65 ± 1.4	1.12 ± 0.8	-4.329	<0.001
Moderate-to-vigorous physical activity time (hours/day)	0.90 ± 0.6	1.38 ± 0.9	6.241	<0.001
Sedentary time (hours/day)	4.50 ± 2.8	3.70 ± 3.3	-2.493	0.013
Time on the roadside where children might be exposed to automobile exhaust (min/day)	14.37 ± 13.3	11.20 ± 8.9	-2.586	0.010
Proportion of plastic bottled water to total drinking water (%)	29.36 ± 39.4	11.81 ± 26.0	-4.830	<0.001
Sleeping information				
Wakeup time in the morning (AM)	7.07 ± 0.7	7.05 ± 0.5	-0.206	0.837
Bedtime at night (PM)	9.77 ± 0.6	9.65 ± 0.6	-1.905	0.057
Night sleeping time (hours/day)	9.30 ± 0.8	9.40 ± 0.6	1.389	0.166
Day sleeping time (min/day)	24.17 ± 33.2	51.83 ± 25.4	8.733	<0.001
Total sleeping time (hours/day)	9.70 ± 1.0	10.26 ± 0.7	5.861	<0.001

The t-test was used to compare the continuous variables and the results were presented in mean ± SD.

The χ^2^ test was used to compare the categorical variables and the results were presented as frequency and percentage (%).

^*^Overweight was defined as BMI ≥ the 85^th^ percentiles for age- and sex-specific distribution according to WHO age- and sex-specific growth reference.

### 3.2 Prediction model based on NGRS

Factors that were statistically correlated with EPD in univariate analyses as shown in **Table 2** were incorporated into multivariate logistics regression using the stepwise method to select the potential influencing variables. Overweight (OR=2.74), longer electronic screen time (OR=1.79) and higher ratio of plastic bottled water (OR=1.01) were potential risk factors, and longer exercise time (OR=0.51) and longer day sleeping time (OR=0.97) were protective factors for EPD. As shown in **Table 3** (Model 1), the AROC of NGRS model was 83.6% (79.3-87.9%).

**Table 3:** Odds Ratios (95% CIs) and area under receiver operating characteristics curve for genetic risk score, non-genetic risk score and combined risk score models.

	GRS	Model 1		Model 2	
		NGRS	CRS	NGRS	CRS
Potential affecting factors					
Mother’s age of menarche (years)		–	–	0.62 (0.48-0.80)	0.64 (0.49-0.82)
Father’s age of first spermatogenesis (years)		–	–	0.74 (0.58-0.95)	0.75 (0.58-0.96)
Overweight	–	2.74 (1.58-4.76)	2.43 (1.38-4.28)	2.37 (1.33-4.21)	2.09 (1.16-3.77)
Moderate-to-vigorous physical activity time (hours/day)	–	0.51 (0.36-0.72)	0.47 (0.33-0.68)	0.52 (0.37-0.75)	0.49 (0.33-0.71)
Electronic screen time (hours/day)	–	1.79 (1.37-2.33)	1.86 (1.42-2.43)	1.75 (1.34-2.28)	1.83 (1.40-2.41)
Day sleeping time (min/day)	–	0.97 (0.96-0.98)	0.97 (0.96-0.98)	0.97 (0.96-0.98)	0.97 (0.96-0.98)
Proportion of plastic bottled water (%)	–	1.01 (1.01-1.02)	1.01 (1.00-1.02)	1.02 (1.01-1.02)	1.02 (1.01-1.02)
GRS	1.90 (1.47-2.46)	–	2.07 (1.51-2.85)	–	2.06 (1.48-2.88)
AROCs and 95% CIs	0.653 (0.597-0.708)	0.836 (0.793-0.879)^*^	0.857 (0.817-0.896)^*^	0.861 (0.821-0.901)^#^	0.874 (0.836-0.912) ^#^

Model 1, without mother’s first menarche age or father’s first spermatogenesis age; Model 2, with mother’s first menarche age and father’s first spermatogenesis age.

DeLong’s test for the difference between AROCs of NGRS model and CRS model: ^*^Z = -2.33, P=0.020; ^#^Z = -1.78, P=0.074. '-' means that there was no value to display.

After taking into account the influence of family history (Model 2), mother’s first menarche age and father’s first spermatogenesis age were inversely associated with EPD, and the AROC of NGRS model improved [86.1% (82.1-90.1%)].

### 3.3 Prediction model based on GRS

The associations of SNPs with EPD are presented in **Table 1**. The weighted GRS was computed by multiplying the number of risk alleles (0, 1, or 2) of each SNP by the natural logarithm of OR in the Logistic regression for that allele and summing across all SNPs. The mean gene count score was 5.00 (SD 0.83) in girls with EPD and 4.51 (SD 0.84) in girls without EPD. The GRS showed a significant association with EPD (OR=1.90), and the AROC of GRS model was 65.3% (59.7-70.8%) (**Table 3**).

### 3.4 Prediction model based on CRS

**Figure 1:**
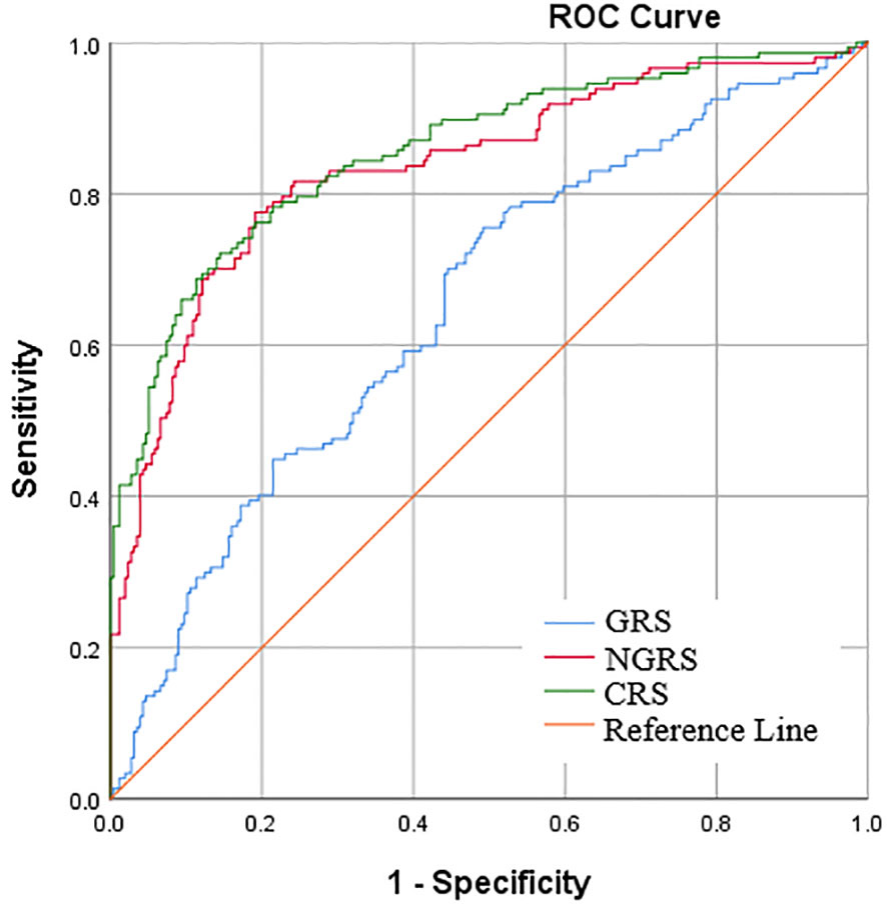
Area under receiver operator characteristic curve (AROC) for early pubertal development. The non-genetic risk score (NGRS) was calculated from 6 biochemical predictors of independent risk (Model 1), and the genetic risk score (GRS) was constructed using 28 EPD related single-nucleotide polymorphisms (SNPs).

CRS was calculated by adding the weighted GRS to the NGRS model to obtain a combined genetic and non-genetic score. In Model 1 analyses, the AROC of CRS significantly increased by 2.1% as compared with the AROC of NGRS model [85.7% (81.7-89.6%) vs. 83.6% (79.3-87.9%), *Z* = -2.33, *P*=0.020] (**Table 3**, **Figure 1**). In Model 2 analyses, the AROC of CRS increased by 1.3% as compared with the AROC of NGRS model [87.4% (83.6-91.2%) vs. 86.1% (82.1-90.1%), *Z* =-1.78, *P*=0.074] (**Table 3**).

### 3.5 Reclassification of CRS

We used NRI to assess the extent to which adding GRS to the NGRS model resulted in the movement of prediction accuracy of EPD. In these analyses, we used the same three categories (< 10%, 10% - <90%, and ≥ 90%) and did the analyses separately for girls diagnosed as having EPD and those without EPD. As shown in **Table 4**, the addition of GRS to NGRS resulted in a NRI of 8.19% (95% CI: 1.11% - 15. 27%, *P*= 0.023) in the categorical analysis, and of 50.75% (95% CI: 31.15% - 70.35%, *P <*0.001) in the continuous analysis. The IDI was also calculated to reflect the extent to which adding GRS to the NGRS model resulted in the movement of prediction accuracy of EPD, and it was 4.22% (95% CI: 2.20% - 6.23%, *P <*0.001).

**Table 4:** NRI and IDI based on addition of GRS to NGRS, calculated using risk cutoffs of 10% and 90%.

Predicted risk by NGRS	Number of participants			Net reclassified (%)
	Low risk [0, 10%)	Middle risk [0%, 90%)	High risk [90%, 100%]	
Plus externally weighted gene score: without EPD (n = 256)				
Low risk [0, 10%)	62	12	0	16
Middle risk [0%, 90%)	26	156	0	14
High risk [90%, 100%]	0	0	0	–
Plus externally weighted gene score: with EPD (n = 147)				
Low risk [0, 10%)	5	1	0	20
Middle risk [0%, 90%)	3	107	8	10
High risk [90%, 100%]	0	2	21	14
Plus externally weighted gene score: combined data (n = 403)				
Low risk [0, 10%)	66	12	0	16
Middle risk [0%, 90%)	30	264	8	13
High risk [90%, 100%]	0	2	21	14

NRI (Categorical) (95% CI): 8.19% [1.11% - 15.27%], P= 0.023;

NRI (Continuous) (95% CI): 50.75% [31.15% - 70.35%], P <0.001;

IDI (95% CI): 4.22% [2.20% - 6.23%], P <0.001. '-' means that there was no value to display.

## 4 Discussion

Developing a prediction model for identifying individuals at risk is important to formulate measures for preventing or delaying disease onset. To our knowledge, there are no risk prediction models for EPD developed currently. The present study established a combined predictive model for EPD in girls based on both non-genetic and genetic factors. It also assessed the predictive ability improvement as well as the reclassification of adding genetic variants to the non-genetic risk model.

The timing of normal pubertal onset is influenced by both environmental and genetic factors. It is considered that overnutrition, insufficient exercise (20), insufficient sleep (6), as well as expose to some EDCs (7) are linked to EPD. In this study, we found that longer day sleeping time may be a protective factor for EPD. For adults, daytime nap seems to be beneficial to performance on certain cognitive tasks, and it has been suggested a modest causal association between habitual daytime napping and larger total brain volume using Mendelian randomization (21). For children, clinical observation showed that children who did not nap might be fussier and had shorter attention duration than children who napped regularly (22), but there is no research to date evaluating the association of napping with EPD. According to the Guide of Chinese Sleep Medical Society, sleeping time for children aged 6-8 should be 10-12 hours/day. In our study, night sleeping time of girls in both of the two groups was less than 10 hours/day. After adding day sleeping time, the total sleeping time of girls in the control group was 10.26 ± 0.7 hours/day, but the total sleeping time of girls in the case group was still insufficient (9.70 ± 1.0 hours/day). Although insufficient sleep was suggested a risk factor for EPD (6), the difference of total sleeping time between the two groups was not significant. Until now, the mechanism of the association of day sleeping time with EPD is not known, and future studies are needed to explore the related mechanism.

Currently, there are no risk prediction models for early pubertal development (EPD) developed worldwide. The present study confirmed that overweight, longer electronic screen time and higher ratio of plastic bottled water were potential risk factors, and longer exercise time and longer day sleeping time were protective factors for EPD. By constructing the NGRS as the sum of the above affecting factors, the NGRS performed well, with an AROC of 83.6%.

A family disease history, in specific cases, not only reflected genetic tendency but also reflected shared environmental and lifestyle factors, even including fetal programming. In fact, researches showed that a complete family history provided a better prediction than SNPs (23), and family history remained a strong, independent, and easily-to-assess risk factor for some diseases (24). The present study found that mother’s first menarche age and father’s first spermatogenesis age were associated with EPD, which was independent of GRS. After adding the above two family history related factors into the model, the AROC of NGRS increased to 86.1%.

Previous studies have examined associations between certain SNPs and EPD (6, 7). However, such SNPs individually have low predictive ability for the risk of EPD. The GRS provides an opportunity to evaluate the cumulative effects of genetic factors. The present study showed a low effect (OR: 1.00-1.68) of per individual allele, but combining the markers could predicted greater risk (OR≈2). The GRS utilized herein had an AROC of 65.3%.

While the GRS had a lower predictive ability for EPD compared to NGRS, combining both factors prompted a modest increase (2.1% or 1.3% for models without or with family history related factors, respectively). Combining GRS and NGRS resulted in the movement of prediction accuracy of EPD, with NRI of 8.19% and IDI of 4.22%. Specific information for EPD predictive models remained scarce, so we cannot compare our results with others. When compared with type 2 diabetes prediction models, the results of our prediction models were reasonable. The predictive models for type 2 diabetes showed that inclusion of genetic biomarkers resulted in a slight improvement, with differences in AUC ranging from 0 to 12% and NRI from -2.2% to 10.2% (25).

Although the risk classification of EPD has been improved after adding GRS, the moderate impact needs to be considered, and there is not enough evidence to suggest that GRS should be included in clinical practice. However, genetic risk factors remain unchanged throughout the course of life, and the cost of genotyping is much higher than that of conventional risk factors. The conventional risk factors provide a nice predictive value of EPD risk classification, and in most cases, it can be easily obtained only by a medical history, physical examination and a questionnaire. Thus, we highlighted that conventional risk factors, such as family history related factors, overweight, adverse lifestyle, should be considered a priority into clinical practice.

One of the limitations of this study is that our results may not be extended to people of different ethnic backgrounds. Secondly, the cross-sectional design limited the ability to determine the influence of affecting factors on the progression of EPD over time. Thirdly, we could not confirm the relationship between dietary habits and EPD risk, since the nutritional self-management for girls in the case group was established at the time of or even before diagnosis.

## 5 Conclusion

In conclusion, the current study established a combined prediction model of EPD in girls. Adding genetic variants to the non-genetic risk model brought modest improvement. However, the non-genetic factors such as overweight and living habits have higher predictive utility. We emphasize that priority should be given to preventing environmental exposure over unchangeable genetic factors.

## Data availability statement

The original contributions presented in the study are included in the article/supplementary material. Further inquiries can be directed to the corresponding authors.

## Ethics statement

The studies involving humans were approved by Ethics Committee of Tianjin Women and Children’s Health Center. The studies were conducted in accordance with the local legislation and institutional requirements. Written informed consent for participation in this study was provided by the participants’ legal guardians/next of kin.

## Author contributions

WqL: Conceptualization, Data curation, Methodology, Writing – original draft. YD: Conceptualization, Investigation, Methodology, Writing – review & editing. LF: Investigation, Project administration, Writing – review & editing. PS: Investigation, Methodology, Writing – review & editing. LW: Investigation, Methodology, Writing – review & editing. SZ: Investigation, Methodology, Writing – review & editing. WL: Investigation, Methodology, Writing – review & editing. DZ: Conceptualization, Methodology, Supervision, Validation, Writing – review & editing. HL: Conceptualization, Methodology, Supervision, Validation, Writing – review & editing.
